# Prediction Model for Dry Eye Syndrome Incidence Rate Using Air Pollutants and Meteorological Factors in South Korea: Analysis of Sub-Region Deviations

**DOI:** 10.3390/ijerph17144969

**Published:** 2020-07-10

**Authors:** Jong-Sang Youn, Jeong-Won Seo, Wonjun Park, SeJoon Park, Ki-Joon Jeon

**Affiliations:** 1 Department of Environmental Engineering, Inha University, Incheon 22212, Korea; jongsang@inha.ac.kr; 2 Department of Ophthalmology, Hallym University, Dongtan Sacred Heart Hospital, Hwaseong-si 18450, Korea; virgo901@hallym.or.kr; 3 School of Industrial Management Engineering, Korea University, Seoul 02841, Korea; choas6604@naver.com; 4 Division of Energy Resources Engineering and Industrial Engineering, Kangwon National University, Chuncheon-si 24341, Korea

**Keywords:** dry eye syndrome, air pollutants, meteorological factors, prediction model, regional deviation

## Abstract

Here, we develop a dry eye syndrome (DES) incidence rate prediction model using air pollutants (PM_10_, NO_2_, SO_2_, O_3_, and CO), meteorological factors (temperature, humidity, and wind speed), population rate, and clinical data for South Korea. The prediction model is well fitted to the incidence rate (R^2^ = 0.9443 and 0.9388, *p* < 2.2 × 10^−16^). To analyze regional deviations, we classify outpatient data, air pollutant, and meteorological factors in 16 administrative districts (seven metropolitan areas and nine states). Our results confirm NO_2_ and relative humidity are the factors impacting regional deviations in the prediction model.

## 1. Introduction

Dry eye syndrome (DES) is a multifactorial disease of the tear film and eye surface that causes eye discomfort and visual impairment [1,2,3,4,5]. DES is one of the world’s most common chronic diseases [6]. Recently, epidemiological studies have reported an increasing incidence rate of DES ranging between 4.3 and 73.5% in the worldwide population [7,8,9,10,11]. Additionally, symptoms of DES have a negative effect on eyesight and quality of life [12], and it is known that various factors of the tear layer and the surface of the eyeball act in combination with the prevalence of DES [13].

The risk factors consistently associated with DES in epidemiologic studies are age, female Gender, postmenopausal estrogen therapy, omega-6 fatty acid level, refractive surgery, antihistamine use, vitamin A deficiency, connective tissue disease, and bone marrow transplantation [14]. Additionally, long-term computer use may reduce eye blinking and cause symptoms including burning, stiffness, redness, and blurring of the eyes [15]. Moreover, 50–75% of contact lens wearers complain of eye irritation [16,17,18]. This may worsen moisture loss and cause tear osmotic pressure and changes in the eye surface, which can be a major risk factor for DES [19]. Air pollutants coming into contact with the eyes can have a similar effect on the outbreak of DES. 

Since air pollution has emerged as an international concern, research on its effects on the human body is underway. Since air pollutants cause eye diseases [20,21,22], the association between air pollution and DES is of particular interest. Studies have reported that ocular surface damage caused by air pollutants causes tear film instability which causes DES [23,24,25]. Two case studies have reported significant relationships between air pollution and DES prevalence in South Korea [26,27]. Um et al. observed a correlation between DES and atmospheric SO_2_ concentrations and reported DES onset according to the degree of urbanization [26]. Hwang et al. analyzed the correlation between O_3_ level and DES and found that high O_3_ concentrations and low humidity were associated with the occurrence of DES among Koreans [27]. Since the correlation between environmental factors and DES has been clarified through epidemiological researches in South Korea, it is necessary to develop a prediction model for DES incidence caused by air pollution so the model contributes to the public health perspective.

During this study, we analyze the air pollutants and meteorological factors associated with DES incidence rate data. Furthermore, we develop a nationwide DES incidence rate prediction model and analyze regional deviations in the DES incidence rate prediction model.

## 2. Materials and Methods 

South Korea is divided into 17 administrative districts including Sejong which was newly added in 2012 as a 17th district. However, since this study used environmental and hospital data from 2002 to 2015, Sejong was excluded to avoid statistical error. 

### 2.1. Enviromental Data

**Figure 1:**
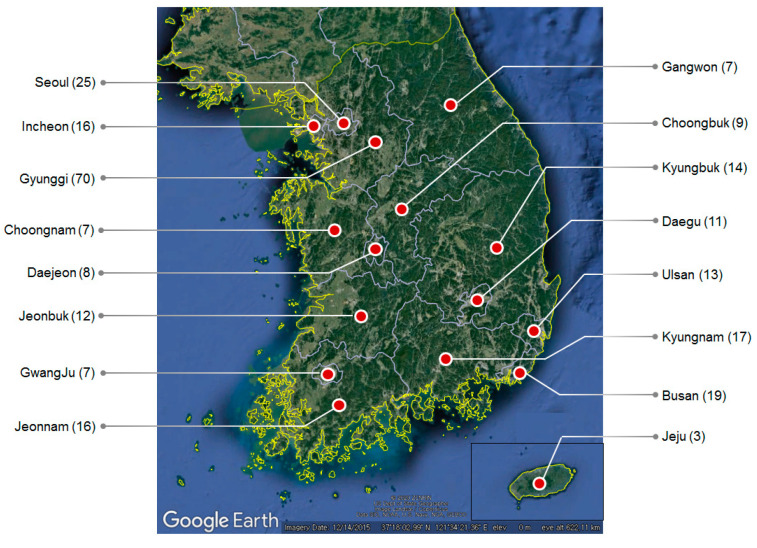
Number of air pollutant and meteorological factor sampling stations for each administrative district (total 254).

Air pollutant (PM_10_, NO_2_, SO_2_, O_3_, and CO) levels and meteorological factors (temperature, relative humidity, and wind speed) measured hourly from 1 January 2002, to 31 December 2015, were retrieved from a total of 254 air pollution monitoring networks operated by the Korean government. Each administrative district has from 3 to 70 sampling stations depending upon the regional population density (Figure 1). According to the population statistics in 2019, half of the Korean population is concentrated in the metropolitan area (Seoul, Incheon, and Gyunggi), so about 44% of sampling stations are in the metropolitan area. The hourly measured meteorological factors were obtained adjacent to the sampling sites. We then calculated the monthly average of nationwide and 16 administrative divisions in South Korea.

### 2.2. Dry Eye Syndrome Hospitalization Data

DES hospitalization data between January 2002 and December 2013 in South Korea was provided by the Korea Health Insurance Review and Assessment Service (KHIRAS) for research purposes. Since South Korea operates public health insurance, hospitals are required to report patient medical records. A total of 48,344 DES patient data were obtained, based on disease code. The ophthalmological outpatient data were classified based on diagnostic codes after removing patient personal information by the KHIRAS. The National Health Insurance Service (NHIS) of South Korea provides public health insurance for 95% of South Koreans [28], and hospitals in South Korea are required to submit medical service documents to NHIS. It was able to classify DES outpatient data by administrative districts because the hospital data includes not only patient disease codes but, also, the information of the administrative districts. Population data from 2002 to 2007 and from 2008 to 2013 were obtained from Statistics Korea and the Korean Ministry of the Interior and Safety, respectively. 

### 2.3. Dry Eye Syndrome Incidence Rate Prediction Model

Regarding model development, the monthly average of environmental and DES outpatient data was used. DES outpatient data and environmental data were provided on a daily basis and on an hourly basis, respectively, so the environmental data were reproduced on a daily average. However, a daily average-based prediction model might have been affected by the weekend and holiday data, so it was necessary to add variables related to the day of the week. To avoid adding the day of the week variables, it was appropriate to analyze the data on a weekly or monthly basis. Our previous study used data for 3 years, so the weekly average was used because the number of data was very small when using a monthly average [29]. However, since this study used statistical data from 2002 to 2015, monthly average data was suitable to reduce errors caused by delaying hospital visits due to weather conditions and other factors. Further, using the monthly average was more appropriate than using the annual or weekly averages for the analysis of the seasonal effects on DES incidence. The DES incidence rate means the number of DES outpatients divided by the population (Equation (1)). Nationwide and regional DES incidence rates were calculated separately.

(1) $$Incidence\;rate=\frac{The\;number\;of\;outpatients}{Population}$$

where, the number of outpatients is the number of patients who have been diagnosed with DES by visiting a hospital, and the population is the number of people who live in the administrative districts. Correlation analysis was first conducted using nationwide monthly averages of DES incidence rates, air pollutant levels, and meteorological factors to identify which of these factors most significantly affected the DES outpatients. Then, based on the results of the analysis, a general regression model was used to develop a nationwide DES incidence rate prediction model. Finally, we used the same procedure to develop models for the 16 administrative divisions.

## 3. Results and Discussion

### 3.1. Monthly Average of Dry Eye Syndrome Incidence Rates, Air Pollutant Levels, and Meteorological Factors

**Figure 2:**
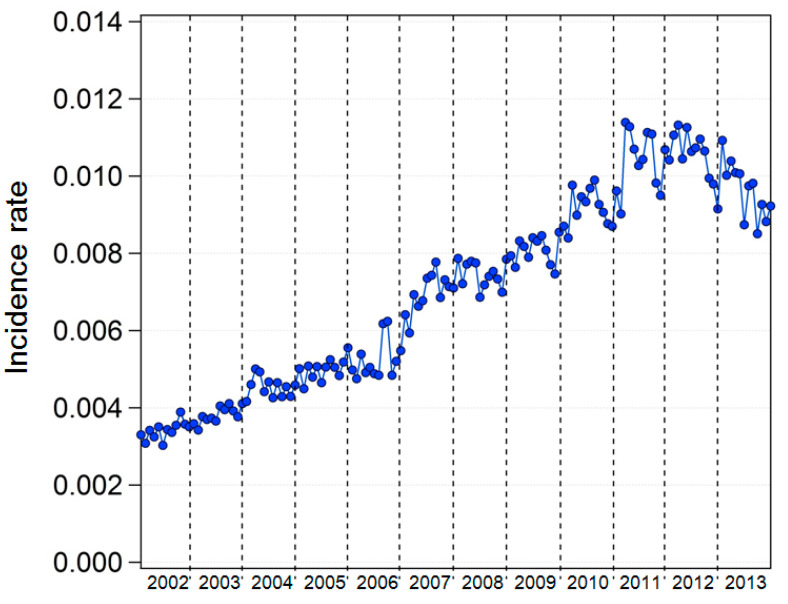
Monthly average DES incidence rates, 2002–2013.

The nationwide monthly average DES incidence rates increased sharply from 2002 to 2011 but decreased from 2012 to 2013 (Figure 2). 

**Figure 3:**
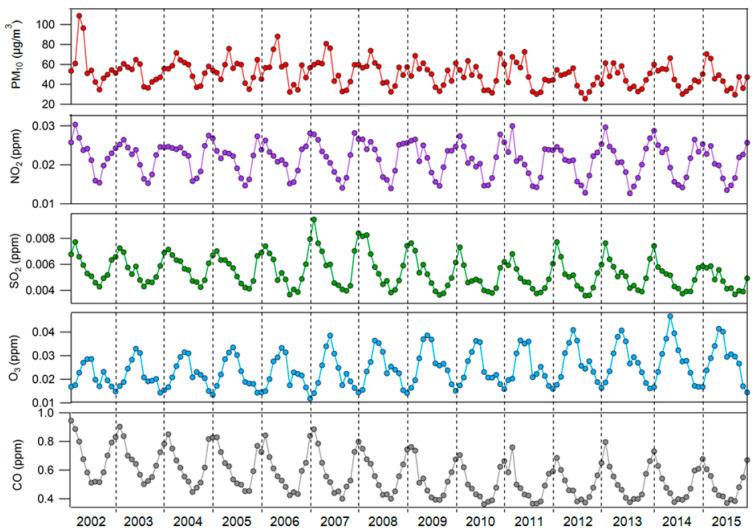
Monthly average air pollutant concentrations, 2002–2015. Figure 4 shows the monthly averages of meteorological factors (temperature, relative humidity, and wind speed) from 2002 to 2015. Temperature and humidity were highest in the summer months (June–September) and lowest in the winter months (December–February). Wind speed differed from year-to-year but was generally high between February and April.

Figure 3 shows the monthly average PM_10_, NO_2_, SO_2_, O_3_, and CO levels. PM_10_ showed high concentrations in March–May and mainly a low concentration in July–September. O_3_ concentrations were highest in April–May and the lowest in November–January. CO, NO_2_, and SO_2_ showed the highest and lowest concentrations during the winter and summer months, respectively.

Air pollutants (PM_10_, NO_2_, SO_2_, O_3,_ and CO) and meteorological factors (temperature, relative humidity, and wind speed) were normalized to Equation (2) for correlation analysis.

(2) $$Normalized\;data=\frac{Original\;data-\mu }{\sigma }+4$$

where *µ* and *σ* are the mean and standard deviation of the original data, respectively. The z-score is defined by Equation (3) and provides a standardized factor for how far the data are from the *σ* value. To facilitate data analysis, 4 was added to Equation (2) to remove negative values. Table 1 shows the variables for each category used in model development. The DES incidence rate is designated as *y*. Air pollutants and meteorological factors are designated as x1–5 and z1–3, respectively. Population rate, another variable used in the correlation analysis, is the ratio of each Gender population to total population and is designated as M1–9 and W1–9. Additionally, M and W refer to the population rate of Men and Women, respectively.

(3) (Original data−μ)/σ

Table 2 shows the results of the correlation analyses between air pollutants, meteorological factors, and DES incidence rates from 2002 to 2013 using the monthly average of nationwide data. PM_10_ was positively correlated with NO_2_ (0.616), SO_2_ (0.569), CO (0.506), and wind speed (0.429) and negatively correlated with temperature (−0.447) and relative humidity (−0.643). The DES incidence rate showed significant positive correlations only with O_3_ (0.274) and a negative correlation with CO (−0.477). These findings are similar to those reported from the analysis of 3 years of data by Hwang et al. [27].

O_3_ did not directly affect the DES incidence rate because the average O_3_ monthly concentrations and the number of DES outpatients tended to increase together until 2011. To verify this observation, correlation analyses of DES incidence rates were performed using regional data for each year (2002 ~ 2013) using the averages of each factor from the 16 administrative districts (Table 3). Districts with positive and negative correlation coefficients for x1–z3 indicated high and low DES incidence rates, while those with low negative correlation coefficients for x1–z3 indicated low DES incidence rates. Correlation analyses by year in consideration of regional deviations, air pollutants, and meteorological factors correlated with DES incidence rates differed from the nationwide results, as shown in Table 2. PM_10_, NO_2_, and SO_2_ concentrations were positively correlated with DES incidence rates except in 2003 and 2010, while relative humidity was negatively correlated with DES incidence rates. This is the result of excluding yearly deviations; thus, it was necessary to study other factors that could explain yearly deviations. Since the correlation analyses using nationwide average data in Table 2 was not analyzed by year, it showed a pattern of increasing O_3_ and DES incidence rates according to year, indicating that this was not an accurate result. However, it was still informative to analyze the correlations between each air pollutant and the meteorological factors.

**Figure 5:**
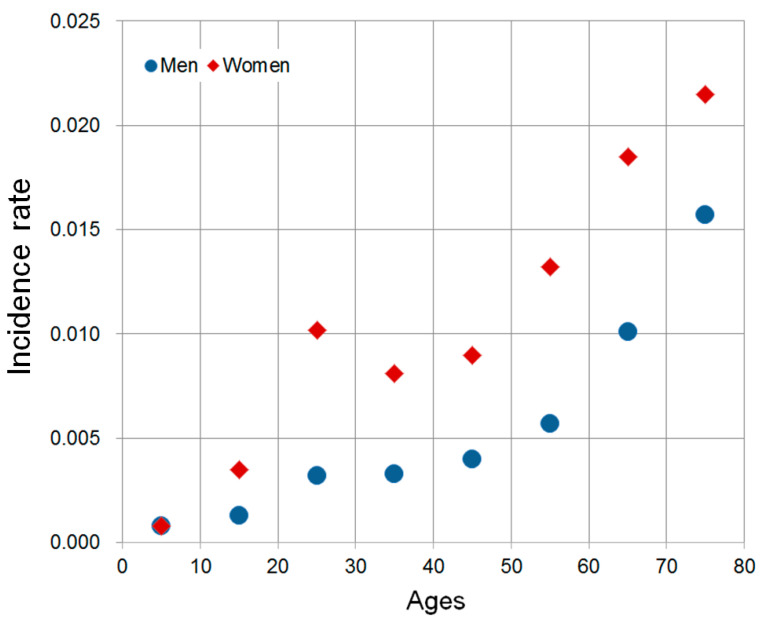
Nationwide DES incidence rates by Gender and age ranges.

Figure 5 shows the DES incidence rates by Gender and age ranges, which shows significant associations with DES incidence rates. A steady increase was observed in men from their 10s to 80s. Concerning women, the DES incidence rate increased in their 20s, decreased slightly from their 30s to 40s, and then increased in their 70s. The incidence rates for women were higher than those in men in all age ranges.

The nationwide data show how each variable affected the annual deviations without considering regional deviations, while the regional data show how regional deviations affected DES incidence rates. Thus, the absolute value of the correlation will be high if the difference in population rate by year and region directly affects the DES incidence rate. Table 4 summarizes the results of the correlation analyses of DES incidence rates in nationwide and regional data by Gender and age ranges. The highest correlation coefficient of M (or W) (0.898 or −0.898) shows that changes in population rate by Gender significantly affected the DES incidence rate. M1 and W1 shows the highest negative correlation coefficients in both nationwide (M1: −0.954; W1: −0.957) and regional (M1: −0.744; W1: −0.739) analyses, while M6 and W6 have the highest positive correlation coefficients in both national (M6: 0.952; W6: 0.952) and regional (M6: 0.822; W6: 0.806) analyses, indicating their significant impacts on DES incidence rates.

### 3.2. Dry Eye Syndrome Incidence Rate Prediction Model

Based on the results of the correlation analyses, we developed a nationwide DES incidence rate prediction model using the population rate for all Men, M, the 10s, MW1 (M1 and W1), and 60s, MW6 (M6 and W6) age ranges as model variables. To minimize independent factors, M1 and M2, M6 and W6 were each combined into one independent variable. M was used to indicate the difference between Men and Women. Model 1 shows the DES incidence rate prediction model using general linear regression. Added to the Gender and age factors, x1–3 (PM_10_, NO_2_, SO_2_) and z_2_ (RH), which are considered factors related to DES incidence rates, also were tested; x1–3 was not significant for Model 1; among them, z_2_ was the most appropriate variable. The following equation indicates the nationwide DES incidence rate prediction model. Model 1:

(4) y=−1.817+3.644×M−0.129×MW1+0.09439×MW6−0.00001139×z2

Considering 2002–2013, two-thirds of the data were randomly selected for the development of Model 1 (in-sample); the remaining one-third of the data were used to validate Model 1 (out-of-sample). Table 5 summarizes Model 1 test results. The results of Model 1 validation showed a statistically significant *p*-value (<2.2 × 10^−16^) for DES incidence rate prediction. The in-sample and out-of-sample R^2^ values (0.9443 and 0.9388, respectively) were both above 93%, indicating that Model 1 was appropriate for predicting the DES incidence rate.

**Figure 6:**
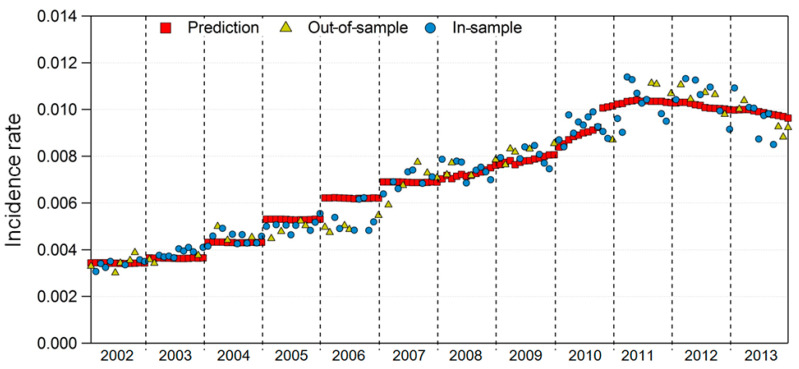
In-sample and out-of-sample incidence rates versus prediction in Model 1.

Figure 6 shows the in-sample and out-of-sample DES incidence rates of prediction using Model 1. The prediction results show similar tendencies between in-sample and out-of-sample incidence rates.

To assess regional deviations in the DES incidence rate model, we categorized the data into 16 administrative districts based on area codes (Table 6). South Korea was divided into seven metropolitan areas and nine state areas.

Model 2 for regional deviation analysis was developed using the same method as that for Model 1. Correlation analyses considering local deviation (Table 3) tested x1, x2, x3, and z2 as model variables. The variables most significantly affecting regional deviation, x2 and z2, were included as variables in Model 2. Since x2 was positively correlated with x1 and x3 during correlation analyses (Table 2), Model 2 was suitable even if x1 or x3 were used rather than x2; however, x2 was used to develop a more optimized model. Like Model 1, Model 2 also was developed by randomly extracting two-thirds of the data from 2002 to 2013 (in-sample), with the remaining data (out-of-sample) used to validate the model.

Model 2:

(5) y=−0.07266+0.1247×M+0.01715×MW1+0.1228×MW6+0.0001718×x2−0.0001036×z2

Table 7 shows Model 2 test results. Validation of Model 2 showed a statistically significant *p*-value (<2.2 × 10^−16^); the in-sample and out-of-sample R^2^ values (0.7085 and 0.7219, respectively) were over 70%, confirming that Model 2 was suitable for predicting DES incidence rates.

**Figure 7:**
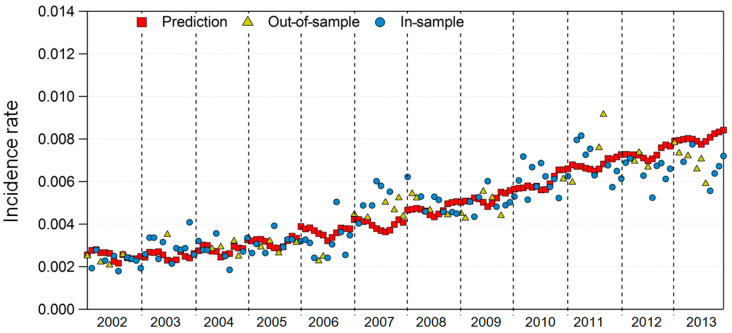
In-sample and out-of-sample incidence rates versus prediction in Model 2 for area 29.

**Figure 8:**
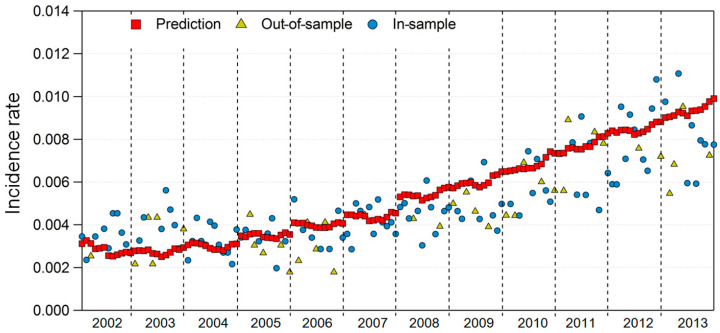
In-sample and out-of-sample incidence rates versus prediction in Model 2 for area 49.

Figure 7 and Figure 8 show the model results for regions 29 and 49, where Model 2 fit the best among the 16 regions. Although not as good as the results of Model 1, the prediction of the DES incidence rate and the in-sample and out-of-sample incidence rates showed similar trends in both regions.

Regarding the case of Model 2, rather than predicting the DES incidence rate, it is significant to verify the variables x2 and z2 that affect regional deviations. When more accurate predictions of DES incidence rates are desired, the data for each region should be used separately to make a prediction model using the same methods used to develop the nationwide model. 

## 4. Conclusions

Here, Model 1 was developed to predict the DES incidence rate from nationwide data. Model 1 confirmed the significant impact of change in population rate and Gender-to-population ratios on the DES incidence rate. The use of Model 1 may allow for accurate prediction of the DES incidence rate. To analyze regional deviations in the prediction model, air pollutants, meteorological factors, and DES incidence rate data were classified by administrative district, and correlation analysis was performed to analyze the effect of regional deviation on the DES incidence rate. NO_2_ and RH were identified as the factors most influencing regional variation. Model 2 was developed by adopting NO_2_ and RH as variables and was verified in a general regression model. However, these variables alone did not account for all regional deviations and require further study. More accurate incidence rate prediction in each region requires the use of statistical methods in the regional DES incidence rate prediction model.

## Figures and Tables

**Figure 4:**
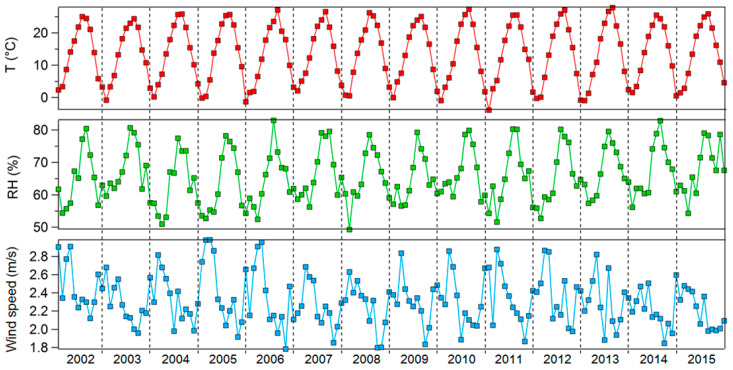
Monthly averages of meteorological factors, 2002–2015.3.2. Correlation Analysis.

**Table 1:** Variables for correlation analysis.

Data	Categories	Variables
DES incidence rate	Incidence rate	*y*
Air pollutant data	PM_10_, NO_2_, SO_2_, O_3_, CO	x1, x2, x3, x4, x5
Meteorological data	Temperature, humidity, wind speed	z1, z2, z3
Population rates	Men: (all), (0–9), (10–19), (20–29), (30-39), (40-49), (50-59), (60-69), (70–79), (over 80) years	Men: M, M1, M2, M3, M4, M5, M6, M7, M8, M9
	Women: (all), (0–9), (10–19), (20–29), (30-39), (40-49), (50-59), (60-69), (70–79), (over 80) years	Women: W, W1, W2, W3, W4, W5, W6, W7, W8, W9

**Table 2:** Correlations among incidence rates, air pollutant data, and meteorological data (nationwide averages).

	x2	x3	x4	x5	z1	z2	z3	y
x1	0.616	0.569	0.183	0.506	−0.447	−0.643	0.429	−0.272
x2	1.000	0.815	−0.324	0.835	−0.841	−0.784	0.185	−0.176
x3		1.000	−0.350	0.876	−0.837	−0.735	0.350	−0.226
x4			1.000	−0.559	0.414	0.035	0.151	0.274
x5				1.000	−0.815	−0.649	0.274	−0.477
z1					1.000	0.833	−0.479	0.025
z2						1.000	−0.574	0.062
z3							1.000	−0.041

**Table 3:** Annual correlations between DES incidence rate and environmental factors considering regional deviation.

	2002	2003	2004	2005	2006	2007	2008	2009	2010	2011	2012	2013
x1	0.488	0.287	0.555	0.373	0.151	0.290	0.232	0.012	−0.034	0.188	0.286	0.147
x2	0.475	0.330	0.400	0.377	0.183	0.344	0.252	0.283	0.296	0.284	0.226	0.154
x3	0.153	−0.069	0.134	0.155	0.036	0.127	0.173	0.290	0.324	0.421	0.262	0.391
x4	0.128	0.049	−0.186	−0.436	−0.260	−0.478	−0.382	−0.395	−0.305	−0.374	−0.146	−0.066
x5	−0.089	−0.017	0.019	−0.112	−0.164	0.075	0.013	−0.088	−0.096	−0.16	−0.381	−0.422
z1	0.119	0.149	−0.066	−0.090	−0.100	−0.024	−0.070	−0.038	0.043	−0.005	0.028	0.241
z2	−0.216	−0.232	−0.311	−0.423	−0.198	−0.309	−0.211	−0.260	−0.360	−0.361	−0.339	−0.477
z3	0.266	0.134	0.081	−0.103	0.024	−0.075	−0.128	−0.052	−0.052	−0.025	−0.122	−0.015

**Table 4:** Correlations between incidence and population rates of nationwide and administrative district by sex and age.

District	M	M1	M2	M3	M4	M5	M6	M7	M8	M9
Nationwide	−0.898	−0.954	−0.565	−0.963	−0.947	0.788	0.952	0.949	0.944	0.920
Administrative district	0.215	−0.744	−0.174	−0.508	−0.390	0.346	0.822	0.249	0.359	0.040
**District**	**W**	**W1**	**W2**	**W3**	**W4**	**W5**	**W6**	**W7**	**W8**	**W9**
Nationwide	0.898	−0.957	−0.616	−0.961	−0.942	0.752	0.952	0.927	0.946	0.935
Administrative district	−0.215	−0.739	−0.304	−0.457	−0.290	0.388	0.806	0.012	0.215	−0.060

**Table 5:** Model 1 test results.

**In-sample test**	R^2^	0.9443
	*p*-value	<2.2 × 10^−16^
**Out-of-sample test**	R^2^	0.9388

**Table 6:** Area codes for each administrative district.

District	Area	Area Codes
Metropolitans	Seoul, Busan, Daegu, Incheon, GwangJu, Daejeon, Ulsan	11, 26, 27, 28, 29, 30, 31
States	Gyunggi, Gangwon, Choongbuk, Choongnam, Jeonbuk, Jeonnam, Kyungbuk, Kyungnam, Jeju	41, 42, 43, 44, 45, 46, 47, 48, 49

**Table 7:** Model 2 test results.

**In-sample test**	R^2^	0.7085
	*p*-value	<2.2 × 10^−16^
**Out-of-sample test**	R^2^	0.7219
